# Pullulan-based films impregnated with silver nanoparticles from the *Fusarium culmorum* strain JTW1 for potential applications in the food industry and medicine

**DOI:** 10.3389/fbioe.2023.1241739

**Published:** 2023-08-07

**Authors:** Magdalena Wypij, Mahendra Rai, Lidija Fras Zemljič, Matej Bračič, Silvo Hribernik, Patrycja Golińska

**Affiliations:** ^1^ Department of Microbiology, Nicolaus Copernicus University in Torun, Torun, Poland; ^2^ Nanobiotechnology Laboratory, Department of Biotechnology, SGB Amravati University, Amravati, India; ^3^ Faculty of Mechanical Engineering, University of Maribor, Maribor, Slovenia

**Keywords:** *Aureobasidium pullulans*, pullulan, nanocomposite films, silver nanoparticles, mycosynthesis, nanobiotechnology, applied microbiology, antibacterial activity

## Abstract

**Introduction:** Biopolymers, such as pullulan, a natural exopolysaccharide from *Aureobasidium pullulans*, and their nanocomposites are commonly used in the food, pharmaceutical, and medical industries due to their unique physical and chemical properties.

**Methods:** Pullulan was synthesized by the *A. pullulans* ATCC 201253 strain. Nanocomposite films based on biosynthesized pullulan were prepared and loaded with different concentrations of silver nanoparticles (AgNPs) synthesized by the *Fusarium culmorum* strain JTW1. AgNPs were characterized by transmission electron microscopy, Zeta potential measurements, and Fourier-transform infrared spectroscopy. In turn, the produced films were subjected to physico-chemical analyses such as goniometry, UV shielding capacity, attenuated total reflection–Fourier-transform infrared spectroscopy, scanning electron microscopy, and X-ray photoelectron spectroscopy, and their mechanical and degradation properties were assessed. The antibacterial assays of the nanoparticles and the nanocomposite films against both food-borne and reference pathogens, including *Listeria monocytogenes, Salmonella infantis, Salmonella enterica, Escherichia coli, Staphylococcus aureus, Pseudomonas aeruginosa*, and *Klebsiella pneumoniae*, were performed using standard methods.

**Results:** AgNPs were small (mean 15.1 nm), spherical, and displayed good stability, being coated with protein biomolecules. When used in higher concentrations as an additive to pullulan films, they resulted in reduced hydrophilicity and light transmission for both UV-B and UV-A lights. Moreover, the produced films exhibited a smooth surface. Therefore, it can be concluded that the addition of biogenic AgNPs did not change the morphology and texture of the films compared to the control film. The nanoparticles and nanocomposite films demonstrated remarkable antibacterial activity against both food-borne and reference bacteria. The highest activity of the prepared films was observed against *L. monocytogenes*.

**Discussion:** The obtained results suggest that the novel nanocomposite films prepared from biosynthesized pullulan and AgNPs can be considered for use in the development of medical products and food packaging. Moreover, this is the first report on pullulan-based nanocomposites with mycogenic AgNPs for such applications.

## 1 Introduction

Currently, many biopolymers are used in different sectors of the food, pharmaceutical, and medical industries due to their unique physical and chemical properties (Rai et al., 2021; Gniewosz et al., 2022). Biopolymers are an alternative to their synthetic counterparts, characterized by biodegradability and high-performance properties (Baranwal et al., 2022). Pullulan, an extracellular biodegradable polysaccharide consisting of repeating units of maltotriose attached by α-(1 → 6) linkages and produced by the polymorphic fungus *Aureobasidium pullulans* (*A. pullulans*), has attracted the attention of many researchers (Rai et al., 2021; Gniewosz et al., 2022; Hernandez-Tenorio and Giraldo-Estrada, 2022; Roy and Rhim, 2023). It is produced under limiting conditions that include media composition, culture conditions (pH, temperature, and time), and the type of *A. pullulans* strains affecting the final fermentation efficiency (Farris et al., 2014; Coltelli et al., 2020; Wei et al., 2020). The resulting pullulan is non-hygroscopic, non-immunogenic, non-mutagenic, non-carcinogenic, tasteless, and odorless (Ran et al., 2017; Coltelli et al., 2020; Rai et al., 2021), exhibits mechanical strength and low viscosity compared to other polysaccharides, and shows functional properties such as adhesiveness, film ability, and fiber formability (Farris et al., 2014; Coltelli et al., 2020). Consequently, pullulan is a unique polysaccharide with many interesting physical and chemical properties, making it a distinctive polysaccharide capable of serving diverse applications. It is identified as generally recognized as safe (GRAS) by the Food and Drug Administration (FDA) in the United States (Rai et al., 2021). Coltelli and coauthors (2020) reported that pullulan has a good measure of flocculating, foaming, and adhesive properties and exhibits antioxidant and prebiotic properties. For this reason, it is increasingly used in dietary fibers. In addition, it can be used as a low-viscosity filler in beverages and sauces (Leathers, 2003). Pullulan is a polymer suitable for use in biodegradable and eco-friendly food packaging and the development of edible coatings due to its good oxygen barrier properties that restrict the growth of aerobic microorganisms and extend the shelf life of food products, water solubility, and non-toxicity (Rai et al., 2021; Wypij et al., 2023). Pullulan-based films are known to play a vital role as remarkable antimicrobial agents against different pathogens (Rai et al., 2021). On the one hand, antimicrobial packaging is a promising form of active food packaging, in particular for meat, fruits, and vegetables, to delay the spoilage process and increase the safety of stored food (Khalaf et al., 2013; Chawla et al., 2021; Duda-Chodak et al., 2023; Wypij et al., 2023).

The pharmaceutical applications of pullulan involve encapsulation of drugs and the drug delivery system, enzyme immobilization, and blood volume expansion, while the medical and biomedical applications of pullulan are associated with controlled drug release, wound healing ability, and anticancer, antimicrobial, and anti-inflammatory activities (Coltelli et al., 2020; Li et al., 2020; Alven et al., 2022).

Polymer dressings based on pullulan possess antimicrobial activity to protect wounds against infections and accelerate their healing (Aderibigbe, 2022; Wypij et al., 2023). This effect can be intensified when some bioactive additives, such as nanoparticles, essential oils, or other biopolymers and bioactive compounds, are incorporated into pullulan-based dressings (Saporito et al., 2017; Kumar et al., 2018; Coltelli et al., 2020; Baron et al., 2022). Among all metallic nanoparticles, silver nanoparticles (AgNPs) are important candidates for solving various medical problems and are also increasingly used in active packaging (Gudikandula et al., 2017). Recently, significant attention has been focused on biogenic AgNPs that possess biocompatibility owing to natural capping in the form of biomolecules, oxidation resistance, and a wide spectrum of antimicrobial activity against pathogens (Gudikandula et al., 2017; Wypij et al., 2022).

**FIGURE 1:**
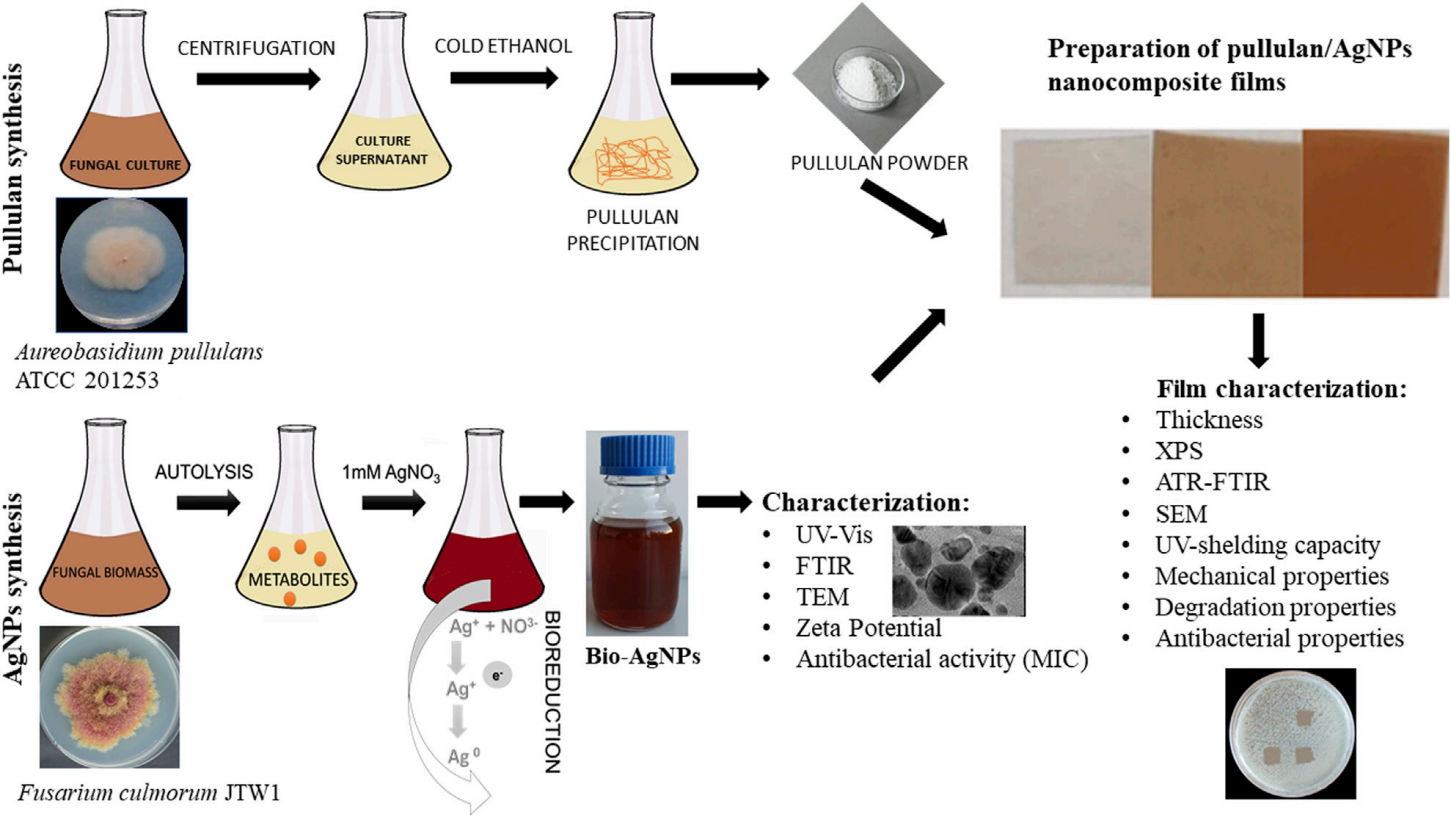
Schema of the present study.

Therefore, this study was designed to synthesize pullulan from *Aureobasidium pullulans* ATCC 201253 and mycogenic AgNPs from the *Fusarium culmorum* strain JTW1 to form novel nanocomposite films for potential applications in food packaging and storage, and medicine. Biosynthesized AgNPs were characterized for physical, chemical, and biological properties using a wide set of techniques, including transmission electron microscopy (TEM), Zeta potential, UV–Vis and Fourier-transform infrared spectroscopy (FTIR), and minimal inhibitory concentration (MIC) against selected Gram-positive (*Staphylococcus aureus* and *Listeria monocytogenes*) and Gram-negative (*Escherichia coli*, *Klebsiella pneumoniae, Pseudomonas aeruginosa, Salmonella infantis*, and *Salmonella enterica*) bacterial strains. In turn, pullulan-based nanocomposites with mycogenic AgNPs were subjected to analyses such as goniometry, UV shielding capacity, attenuated total reflection–Fourier-transform infrared (ATR–FTIR) spectroscopy, X-ray photoelectron spectroscopy (XPS), and scanning electron microscopy (SEM) and evaluations of mechanical and degradation properties. Produced nanocomposite films were evaluated for their antibacterial activity against the bacteria, as mentioned previously. The schema of the present study is shown in Figure 1.

## 2 Materials and methods

### 2.1 Microorganisms

The strain of *A. pullulans* ATCC 201253, used for pullulan biosynthesis, was purchased from the American Type Culture Collection (Manassas, Virginia, United States) and grown on Potato Dextrose Agar (PDA, Becton and Dickinson, United States) for 7 days at 28°C, maintained at 4°C, and sub-cultured at intervals of 2 weeks. For long-term storage, the biomass of *A. pullulans* grown on a PDA medium was maintained at −80°C in 20% (v/v) glycerol.

AgNPs were biosynthesized from the *F. culmorum* strain JTW1, which was isolated and identified, as described previously (Trzcińska-Wencel et al., 2023).

The following pathogens were used for the evaluation of antibacterial activity: food-borne bacteria (*L. monocytogenes* PCM 2191, *S. enterica* PCM 2565*,* and *S. infantis* SES from the Sanitary-Epidemiology Station in Toruń, Poland) and reference bacteria (*E. coli* ATCC 8739, *E. coli* ATCC 25922, *K. pneumoniae* ATCC 700603, *S. aureus* ATCC 25923, *S. aureus* ATCC 6538, and *P. aeruginosa* ATCC 10145). These strains were purchased from the American Type Culture Collection (ATCC; Manassas, Virginia, United States) and the Polish Collection of Microorganisms (PCM; Wrocław, Poland).

### 2.2 Production of pullulan

The basal medium (An et al., 2017), with some modifications, was used for cell growth and pullulan production. The basal medium consisted of starch 50 g (instead of glucose), ammonium sulfate 0.6 g, dipotassium hydrogen orthophosphate 5 g, sodium chloride 1 g, magnesium sulfate 0.2 g, and yeast extract 2.5 g per liter of distilled water, at pH 6.5. The Erlenmeyer flasks containing the basal medium (500 mL) were inoculated with 10 µL of *A. pullulans* culture, grown in the basal medium for 24 h, and incubated for 48 h at 28°C in shaking conditions (120 r.p.m).

Pullulan was precipitated from the culture supernatant according to the previously described method by An et al. (2017) with some modifications. The cultures were centrifuged for 10 min at 10,000 × *g* at 4°C to remove the biomass. The supernatant was transferred into a screw-cap bottle, combined 1:1 (v/v) with absolute cold ethanol, mixed thoroughly, and held for 12 h at 4°C to precipitate the extracellular polysaccharide. Pullulan was separated by centrifugation for 10 min at 8,000 × *g* and 4°C to remove the residual ethanol (An et al., 2017), dissolved in deionized water at 80°C, and purified using dialysis bags (molecular weight cutoff 1 kDa; Spectra/Por^®^6) against deionized water for 72 h to remove small molecules of polysaccharide from the solution (Singh et al., 2012). The exopolysaccharide was precipitated again using cold ethanol, and the residual ethanol was removed by centrifugation for 10 min at 10,000 × *g* and 4°C (Singh et al., 2012). The precipitate was freeze-dried using a lyophilizer (Telstar LyoQuest, Spain), instead of drying at 80°C, and maintained at room temperature for further study.

### 2.3 Preparation of fungal extract for the biosynthesis of AgNPs

The *Fusarium culmorum* strain JTW1 grown on the PDA medium for 7 days at 26°C was cut with a sterile cork borer, and the obtained disk (Ø = 5 mm) was used to inoculate 250 mL of potato dextrose broth (PDB, A&A Biotechnology, Poland) with glass beads in the flask to obtain dispersed mycelial growth. The inoculated flask was incubated for 7 days at 26°C ± 2°C in shaking conditions at 120 r.p.m. The culture was centrifuged for 10 min at 6,000 x *g* at 4°C, the supernatant was discarded, and the cell biomass was washed 3 times with sterile distilled water to remove medium residues and resuspended in sterile distilled water for 3 days for cell autolysis. The autolysate was centrifuged for 10 min at 6,000 x *g* at 4°C, and the obtained supernatant was filtered through sterile Whatman filter paper No. 1. The fungal extract was used for the biosynthesis of AgNPs (Trzcińska-Wencel et al., 2023). The supernatant, water used for biomass washing, and cell residues after autolysation were autoclaved for decontamination before discarding to avoid fungal spread into the environment.

### 2.4 Mycosynthesis of AgNPs

The fungal extract (99 mL) in the glass bottle was treated with 1 mL of 0.1 mol L^-1^ silver nitrate solution (AgNO_3_) to achieve a final concentration of 0.001 mol L^−1^ AgNO_3_ in the reaction mixture, excited in sunlight for 15 min, and kept at room temperature in the dark for 72 h. The pure fungal extract was maintained as a control (Trzcińska-Wencel et al., 2023).

### 2.5 Preparation of pullulan/AgNP nanocomposite film

The thin films were prepared according to the method described by Luís et al. (2020b), with some modifications, using 3% (w/v) of pullulan in 0.75% (w/v) glycerol as a plasticizer. The solution was mixed for 1 h at 80°C using a magnetic stirrer (Hiedolph, Germany).

Pullulan-based nanocomposite films were formed by incorporation of the AgNP solution in deionized water (4 mg mL^-1^) into the pullulan solution (120 mL), which was prepared as described previously. The final concentrations of AgNPs in the preparations were 8, 16, 32, 64, and 128 μg mL^-1^. Preparations were mixed for 5 min at room temperature using a magnetic stirrer and transferred into Petri dishes with a diameter of 90 mm. The pullulan films without AgNPs (control) and nanocomposite films were dried for 24 h at 55°C and subsequently for 3 h at 80°C. Films were stored in a desiccator at 25°C and a relative humidity (RH) of 55% ± 2% (Ferreira et al., 2009).

### 2.6 Characterization of AgNPs

The reduction of metal ions was monitored by visual observation of the color change of the reaction solution from pale yellow to brown.

#### 2.6.1 Ultraviolet–visible spectroscopy

The formation of AgNPs was monitored using ultraviolet–visible (UV–Vis) spectroscopy (NanoDrop ND 2000, Thermo Scientific, Waltham, MA, United States) in a wavenumber range from 280 to 800 nm at a resolution of 1 nm. The sterile distilled water was used as the blank sample, while AgNO_3_ (0.001 mol L^−1^) was used as a control.

#### 2.6.2 FTIR spectroscopy

The FTIR spectrum of biosynthesized AgNPs was recorded using an FTIR Spectrum 2000 instrument (Perkin–Elmer, Waltham, Massachusetts, United States) in the wavelength range of 4,000 cm^−1^–400 cm^−1^ at a resolution of 4 cm^-1^. The sample was prepared in the form of a tablet after combining dried AgNPs with potassium bromide (KBr).

#### 2.6.3 TEM

TEM analysis was performed using an FEI Tecnai F20 X-Twintool Microscope (Fei, Hillsboro, OR, United States), within an accelerating voltage of 100 kV. The AgNP solution in deionized water was dropped on the carbon-coated copper grid (400 μm mesh size) and dried at room temperature before analysis. The obtained data were analyzed using STATISTICA software (StatSoft Inc., Tulsa, OK, United States). The size distribution of AgNPs was estimated based on 180 measurements using TEM imaging and analysis software (TIA).

#### 2.6.4 Zeta potential analysis

Zetasizer (Malvern Instruments Ltd., Malvern, United Kingdom) was used to determine the surface charge of biosynthesized AgNPs, which reflects their stability. All measurements were carried out in triplicate with a temperature equilibration time of 1 min at 25°C. The aqueous suspension of the synthesized AgNPs was 10-fold diluted, homogenized for 15 min at 20 Hz using an ultrasonic homogenizer (Sonic Ruptor 250, Omni Int., Kennesaw, GA, United States) to break down aggregates of nanoparticles, and filtered through a 0.22 μm syringe filter before measurements.

### 2.7 Determination of the minimum inhibitory concentration of AgNPs against food-borne and reference bacteria

The minimum inhibitory concentration (MIC) of AgNPs synthesized from the *F. culmorum* strain JTW1 against bacteria was determined in triplicate using the micro-dilution method in tryptic soy broth (TSB; Becton Dickinson) as a growth medium, according to the method described by the Clinical Laboratory Standards Institute (CLSI). The protocol of the assay was previously described in detail (Wypij et al., 2022; Trzcińska-Wencel et al., 2023). MIC was recorded as the lowest concentration of AgNPs that completely inhibited the visible growth of bacteria after incubation time.

### 2.8 Analysis of the physico-chemical properties of pullulan films

The pullulan-based nanocomposite films prepared from biosynthesized pullulan and AgNPs at different concentrations and control films were analyzed for their physico-chemical properties, as described in the following sections.

#### 2.8.1 Thickness evaluation

The thickness of the film samples was measured using a digital micrometer (BYK-Gardner GmbH, Geretsried, Germany) to the nearest 0.001 mm at five locations in each sample. Measurements were made in triplicate, and the thickness of the films is presented as the mean value.

#### 2.8.2 Surface elemental composition—XPS analysis

To determine the chemical compositions on the surfaces of the samples, the PHI TFA XPS (Physical Electronics, United States) was used. The base pressure in the XPS analysis chamber was approximately 6 × 10^−8^ Pa. The samples were excited with X-rays over a 400 μm spot area with monochromatic Al Kα 1,2 radiation (1,486.6 eV), operating at 200 W. Photoelectrons were detected with a hemispherical analyzer positioned at an angle of 45 with respect to the normal sample surface. The energy resolution was about 0.6 eV. Spectra were recorded for at least two locations on each sample using an analysis area of 400 μm. Surface elemental concentrations were calculated from the survey-scan spectra using MultiPak software.

#### 2.8.3 ATR–FTIR spectroscopy

The spectra were measured using a Spectrum 2000 spectrophotometer (Perkin–Elmer, Waltham, Massachusetts, United States) equipped with a diamond crystal ATR accessory. All the spectra (16 scans) were recorded in the wavelength range of 4,000 cm^−1^–400 cm^−1^ at a resolution of 4 cm^-1^ at room temperature.

#### 2.8.4 Goniometry

The static contact angle (SCA) measurement, which informs on the hydrophilic or hydrophobic nature of the sample, was carried out five times per pullulan film sample using a DataPhysics OCA 35 goniometer (Germany) with SCA 20 software. The film (1 cm × 5 cm) was placed on the base of the sample, and 3 μL of Milli-Q water was applied on the film surface using a microsyringe. The static contact angle of the pullulan films was measured at room temperature.

#### 2.8.5 Mechanical properties

The mechanical test of pullulan-based nanocomposite films enriched with various concentrations of AgNPs and the corresponding control [tensile strength (N/m) and elastic modulus (MPa)] were analyzed using a Shimadzu AG-X plus testing machine with a 10 kN load cell. Tested samples were placed between two clamping clamps and pulled with the upper clamps at a rate of 10 mm/min, following EN ISO 6892-1:2010.

#### 2.8.6 SEM

The morphology of the film samples was studied by SEM (FE-SEM SUPRA 35 VP, Carl Zeiss, Germany) with an accelerating voltage of 5 kV along with a variable working distance and comparable magnification. Films were cut into pieces (0.5 cm × 0.5 cm), mounted in a holder, and sputtered with gold to ensure conductivity and prevent charging effects. The images were acquired using a secondary electron detector.

#### 2.8.7 UV shielding capacity

The transmission spectrum analysis was performed using a Lambda 900 UV–Vis-NIR spectrophotometer (Perkin–Elmer, Waltham, MA, United States) over the UV spectra region of 280 nm–315 nm (UV-B) and 315 nm–400 nm (UV-A). The spectrophotometer analyzed samples with an integrated sphere at a scanning speed of 450 nm/min and a resolution of 5 nm.

#### 2.8.8 Degradation properties

The film samples were cut into small pieces (1 cm × 1 cm), weighted (Wo), placed into small beakers, and soaked with 10 mL in two different solvents, namely, Milli-Q water acidified with HCl to pH 4 and phosphate-buffered saline (PBS; pH 7.4). The beakers were placed on an orbital shaker for different time periods (1 min, 10 min, 30 min, 60 min, and 24 h) at room temperature. The film pieces removed from the solvents were dried at 50°C and weighed to estimate the constant final weight (Wƒ). The degradation index was calculated based on the mass loss using the following formula:

$$\%\;Degradation=\left[\left(Wo-W_{f}\right)/W_{f}\right]\times 100.$$

### 2.9 Antibacterial activity assay of nanocomposites

The antibacterial activity of nanocomposite films was evaluated against food-borne and reference bacteria. The bacterial strains were grown in TSB for 24 h at 35°C ± 2°C under shaking conditions at 120 r.p.m. and used to prepare bacterial inocula in sterile deionized water at a density of 0.5 units on the McFarland scale using a densitometer (Biosan, Latvia). Bacterial inocula were diluted with sterile deionized water to final concentrations of 1–3 × 10^5^ CFU mL^−1^ and spread (100 μL) onto the Tryptic Soy Agar in the Petri plates using a sterile swab. Pullulan films (control) and pullulan-based nanocomposites with ½MIC, MIC, and 2xMIC of AgNPs were aseptically cut into pieces of 1 cm × 1 cm and placed onto the surface of inoculated plates. The plates were incubated at 35°C ± 2°C for 24 h. The zones of growth inhibition under and around the foil pieces were determined in millimeters. The following scale of antibacterial activity was established: zone of inhibition >1 mm around the sample and lack of growth under the sample, very good activity (+++); zone of inhibition < 1 mm and lack of growth under the sample, good activity (++); lack of growth under the sample, low activity (+); and growth under the sample, no activity (−).

## 3 Results

### 3.1 Biosynthesis of AgNPs and their physical and chemical properties

**FIGURE 2:**
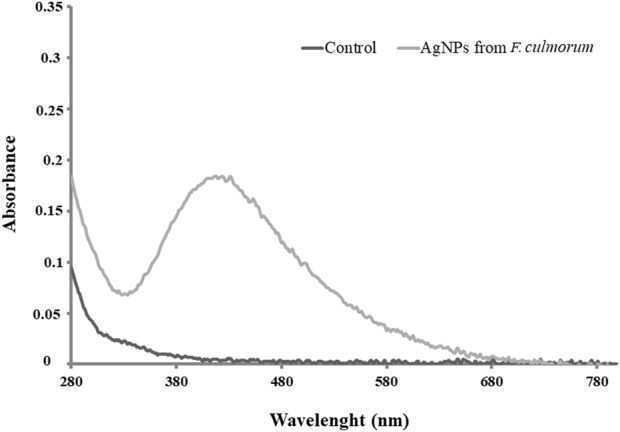
UV–visible spectra of control and AgNPs from the *Fusarium culmorum* strain JTW1.

**FIGURE 3:**
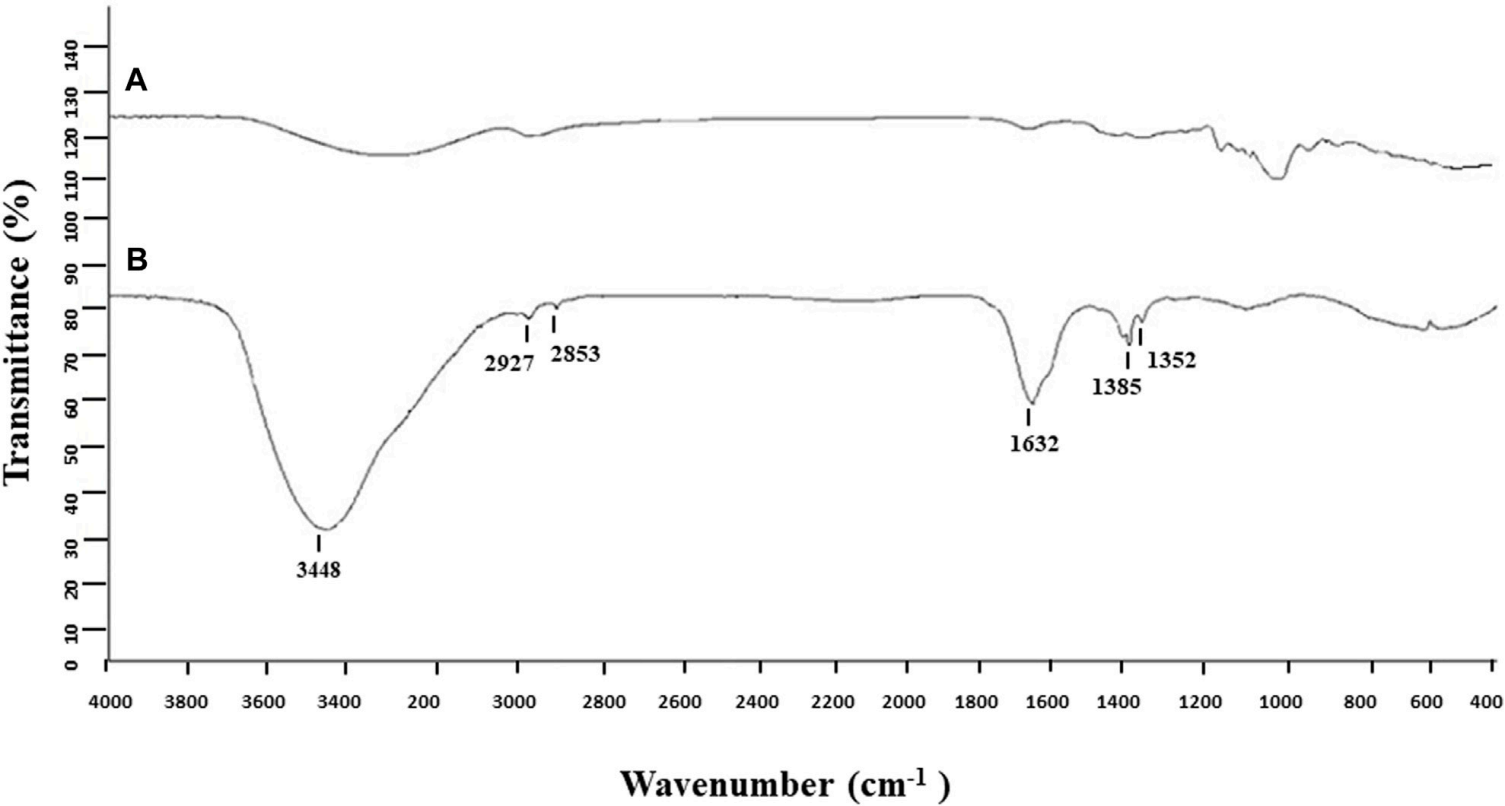
FTIR spectra of control **(A)** and AgNPs from the *Fusarium culmorum* strain JTW1 **(B)**.

**FIGURE 4:**
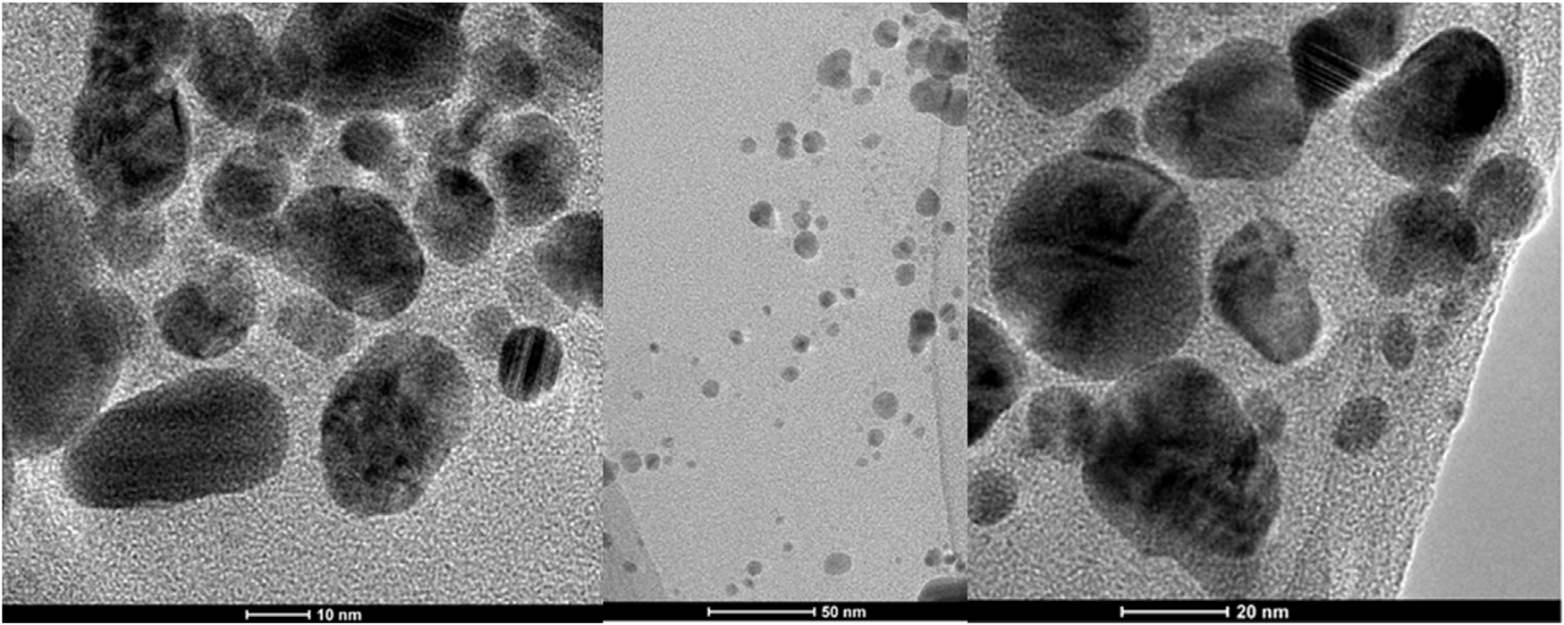
TEM micrographs of AgNPs from the *Fusarium culmorum* strain JTW1.

**FIGURE 5:**
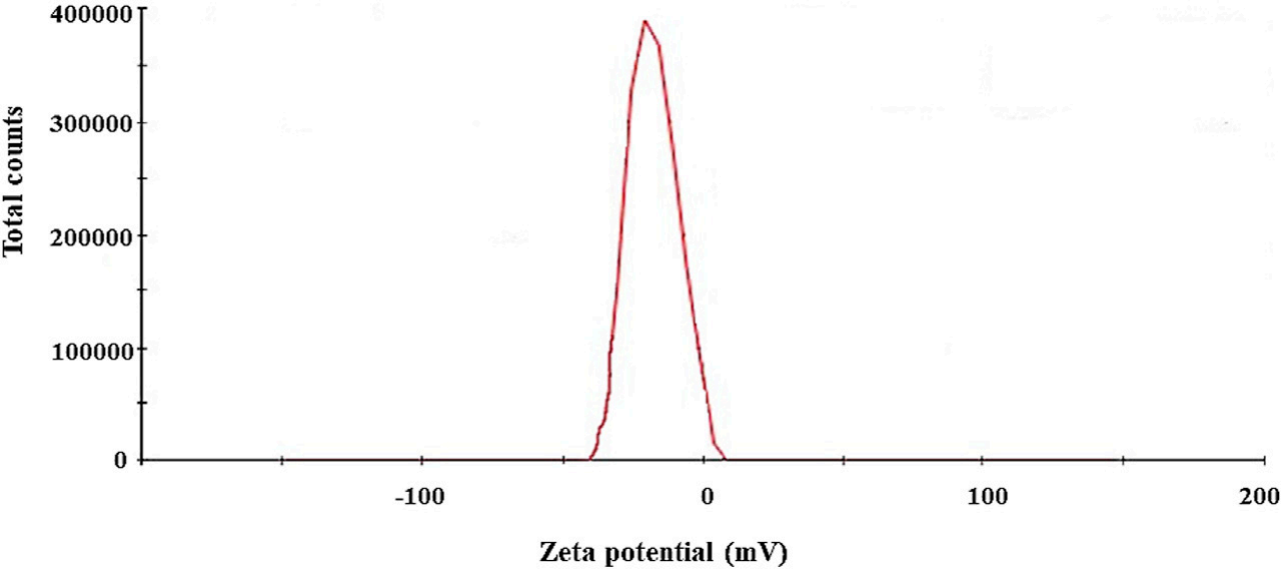
Zeta potential of AgNPs from the *Fusarium culmorum* strain JTW1.

The biosynthesis of AgNPs from the extract of *F. culmorum* strain JTW1 was observed visually by the color change of the reaction mixture from pale yellow to brown when the fungal extract was mixed with AgNO_3_ at a final concentration of 1 mM. The production of AgNPs was also confirmed by UV–visible absorption spectrum scanning in the range of 280–800 nm, which revealed the presence of an absorbance peak at 428 nm (Figure 2). Different functional groups were found in the capping agents of bionanoparticles, as shown in Figure 3. The AgNPs showed an intensive peak at 3,448 cm^-1^, which corresponded to N–H and O–H functional groups; peaks at 2,927 cm^-1^ and 2,853 cm^-1^ were related to alkaline C–H stretching; the peak at 1,632 cm^-1^ corresponded to the C=O (carbonyl group); and peaks at 1,385 cm^-1^ and 1,352 cm^-1^ were related to C–N bonds. TEM images of AgNPs synthesized by the *F. culmorum* strain JTW1 are shown in Figure 4. The TEM analysis showed spherical, well-dispersed, and small AgNPs in the size range of 4–46 nm (Supplementary Figure S1) and a mean size of 15.1 ± 8.2 nm. The zeta potential measurements showed that bionanoparticles capped with biomolecules from the *F. culmorum* strain JTW1 were negatively charged (−30.1 mV) (Figure 5).

### 3.2 Properties of pullulan-based nanocomposite films

**FIGURE 6:**
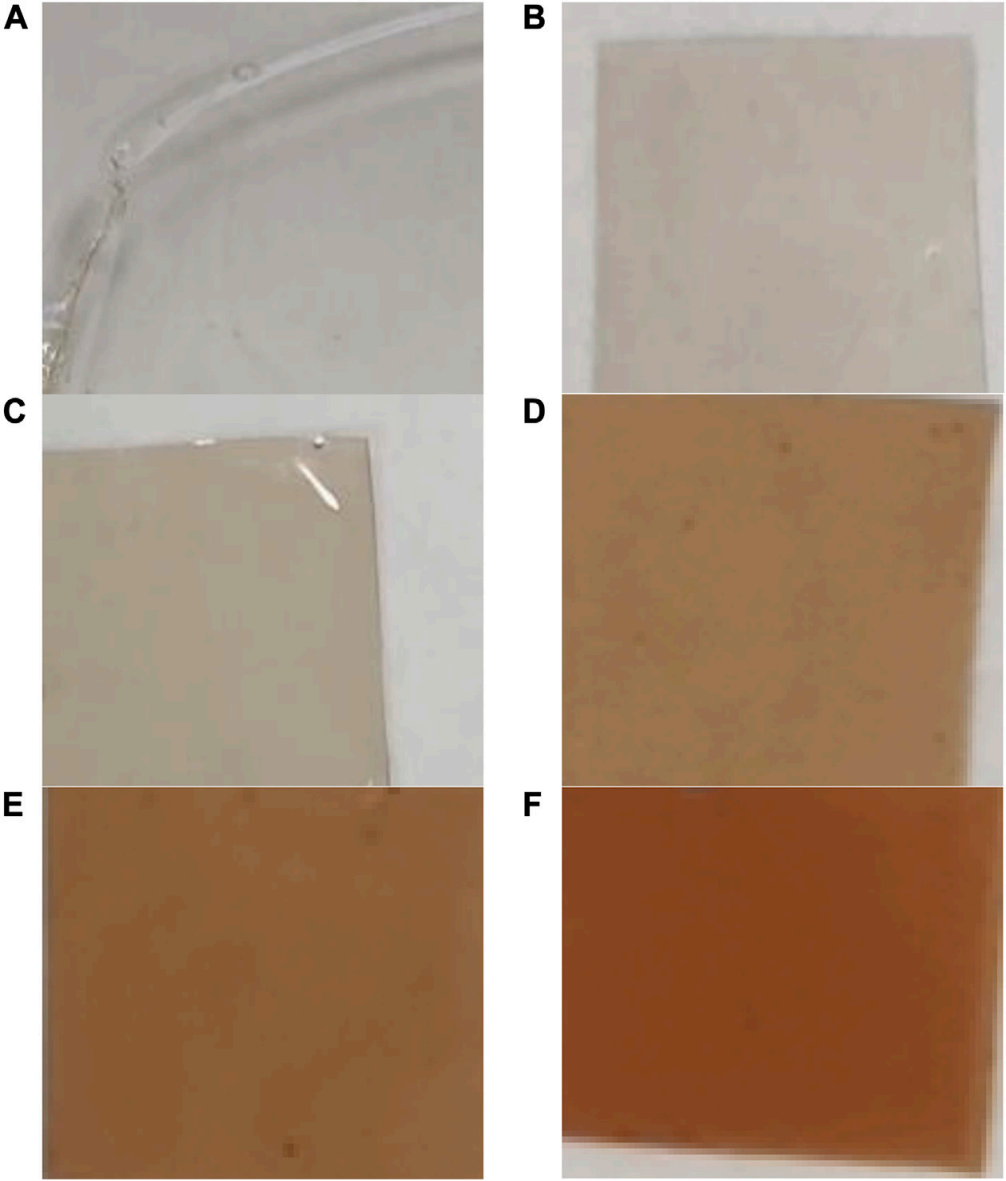
Digital photos of films prepared from pullulan synthesized from *Aureobasidium pullulans* after drying. Control **(A)**, films with AgNPs: 8 μg mL^-1^ **(B)**, 16 μg mL^-1^ **(C)**, 32 μg mL^-1^ **(D)**, 64 μg mL^-1^ **(E)**, and 128 μg mL^-1^ **(F)**.

A change in color intensity of films from colorless (control) to slightly brown was observed with increasing concentrations of AgNPs (Figure 6). The foils with the addition of AgNPs and the control ones were odorless, smooth, and glossy. The control films without AgNP additives were completely transparent.

#### 3.2.1 Thickness and mechanical properties

The thickness of pure pullulan films and pullulan-based nanocomposite foils is shown in Table 1. The thickness of the analyzed films was in the range of 0.03–0.033 mm. The lower values were recorded for the control pullulan film, while the highest values were observed for PUL+64 and PUL+128 μg mL^-1^ AgNPs.

**TABLE 1:** Thickness and mechanical test of pullulan-based nanocomposite films.

Sample	Thickness (mm)	Tensile strength (MPa)	Elastic modulus (MPa)
PUL-control	0.03 ± 4.2	32.9 ± 3.2	600.4 ± 57.3
PUL+8 μg mL^-1^ AgNPs	0.032 ± 2.7	34.9 ± 2.1	685.4 ± 55.4
PUL+16 μg mL^-1^ AgNPs	0.031 ± 3.1	36.3 ± 6.8	1848.2 ± 724.1
PUL+32 μg mL^-1^ AgNPs	0.032 ± 2.1	39.9 ± 9.1	1,529.7 ± 518
PUL+64 μg mL^-1^ AgNPs	0.033 ± 3.3	36.6 ± 4,04	1,498.7 ± 966
PUL+128 μg mL^-1^ AgNPs	0.033 ± 3.4	51.1 ± 9.01	3,090.3 ± 675.9

Results are shown as mean ± SD. PUL, pullulan film.

In the present study, the tensile strength and elastic modulus of the films were determined (Table 1). The tensile strength of pullulan-based nanocomposites ranged from 34.9 MPa to 51.1 MPa, while the elastic modulus ranged from 685.4 MPa to 3,090.3 MPa, depending on the concentration of AgNPs in the films. Overall, the addition of AgNPs generated an increase in both analyzed mechanical parameters compared to the control sample. Pullulan film enriched with the highest AgNP concentration (128 μg mL^-1^) showed the highest elastic modulus (3,090.3 MPa) and tensile strength (51.1 MPa).

#### 3.2.2 X-ray photoelectron spectroscopy analysis

The results of XPS analysis of films made from pullulan synthesized from *A. pullulans* ATCC 201253 and enriched with biologically synthesized AgNPs of various concentrations are shown in Table 2. In the control film, 68.0%, 7.0%, and 25.0% of carbon, nitrogen, and oxygen were found, respectively. Interestingly, films enriched in 128 μg mL^-1^ of AgNPs were characterized by a higher nitrogen content (7.9%) and a lower carbon content (66.1%) when compared to the control sample. However, for pullulan films with the addition of 8 μg mL^-1^, 16 μg mL^-1^, 32 μg mL^-1^, and 64 μg mL^-1^ of AgNPs, the nitrogen content decreased compared to the control sample and was found to be 6.6%, 6.7%, 5.0%, and 4.7%, respectively. Oxygen content was higher in films enriched with 16 μg mL^-1^ and 128 μg mL^-1^ of AgNPs, while the carbon content was higher or equal in most produced films with AgNPs when compared to the control film. Additionally, despite the addition of various concentrations of AgNPs to the films, the XPS analysis showed in each case, including the control, no silver content on the surface (Table 2).

**TABLE 2:** XPS analysis of pullulan films (at%).

Sample	C	N	O	Ag
PUL- control	68.0	7.0	25.0	0
PUL+8 μg mL^-1^ AgNPs	70.2	6.6	23.2	0
PUL+16 μg mL^-1^ AgNPs	68.0	6.7	25.3	0
PUL+32 μg mL^-1^ AgNPs	81.9	5.0	13.1	0
PUL+64 μg mL^-1^ AgNPs	80.2	4.7	15.1	0
PUL+128 μg mL^-1^ AgNPs	66.1	7.9	26.1	0

PUL, pullulan film.

#### 3.2.3 ATR–FTIR spectra analysis

**FIGURE 7:**
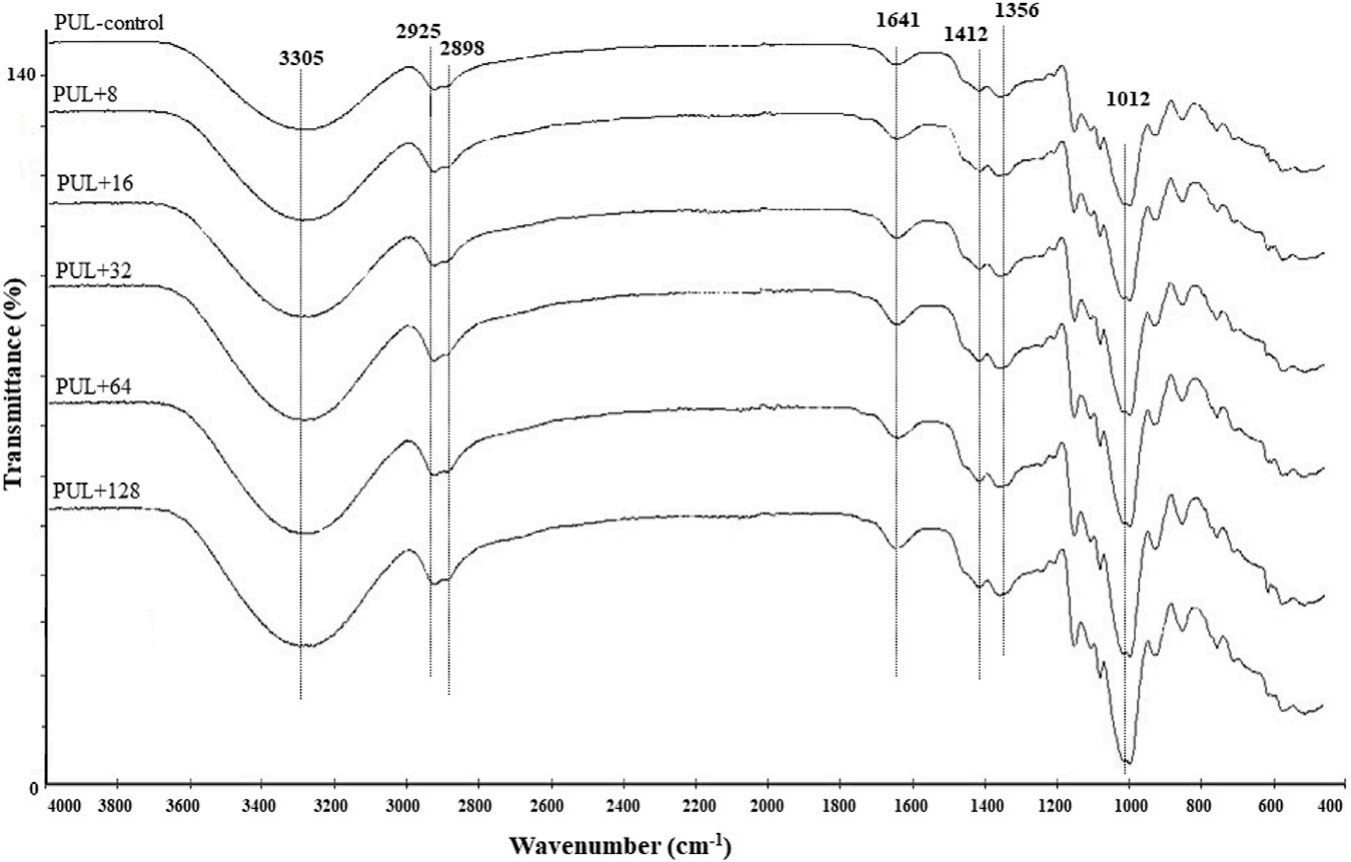
ATR–FTIR analysis of pure film and pullulan films enriched in different concentrations of AgNPs. PUL, pullulan film; 8–128, concentration of AgNPs in μg mL^-1^.

ATR–FTIR spectra were generated to identify potential interactions in the control membranes prepared from synthesized pullulan and their corresponding derivatives enriched in different concentrations of biogenic AgNPs. The films incorporated with AgNPs exhibied a peak at 3,305 cm^-1^, indicating the presence of the hydroxyl (O–H) and amine (N–H) functional groups. Additionally, peaks at 2,925 cm^-1^, 2,898 cm^-1^ and 1.412 cm^-1^ were observed, which corresponded to the C–H streching. Another peak at 1,641 cm^-1^ indicated the presence of the C=O (carbonyl) group, while peak at 1,356 cm^-1^ corresponded to the C–N group. Finally, the peak at 1,012 cm^-1^ was associated with the C–O group (Figure 7).

FTIR analysis of foils prepared from pullulan synthesized by *A. pullulans* revealed intense peaks indicating enhanced adsorption. Additionally, no significant differences were noted between foils incorporated with different concentrations of AgNPs. Moreover, the FTIR analysis showed compliance with the XPS analysis in terms of the elemental composition of the analyzed pullulan films.

#### 3.2.4 Goniometry

**FIGURE 8:**
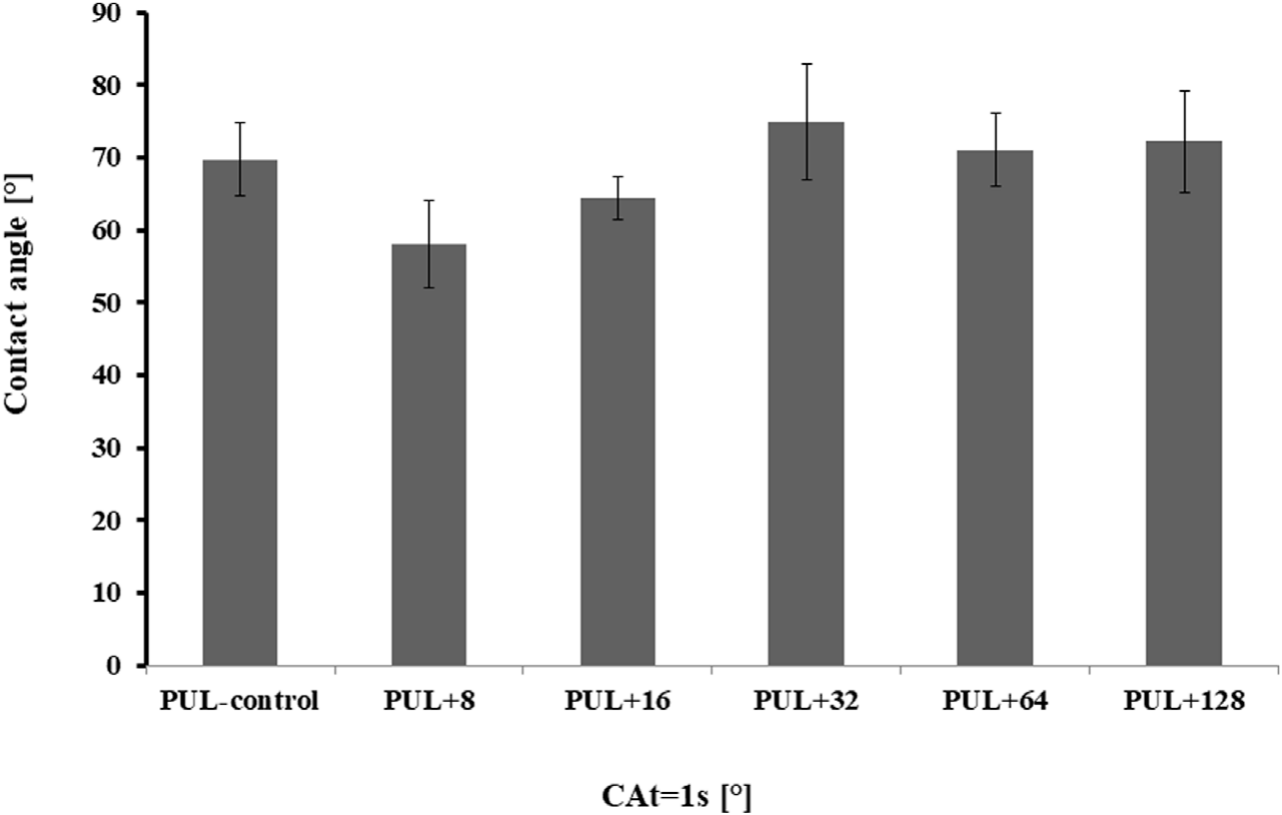
Contact angles of pullulan-based film samples. PUL, pullulan film; 8–128, concentration of AgNPs in μg mL^-1^.

The water SCA of pullulan films is shown in Figure 8. The control pullulan film showed a contact angle of 69.7, indicating the hydrophilicity of the analyzed surface. In contrast, a decrease in the surface hydrophilicity was noted when the films were enriched with 32 μg mL^-1^, 64 μg mL^-1^, and 128 μg mL^-1^ of AgNPs (SCA was equal to 74.9, 71.0, and 72.2, respectively). In the remaining cases, the contact angle was comparable to the control sample or slightly lower.

#### 3.2.5 SEM

**FIGURE 9:**
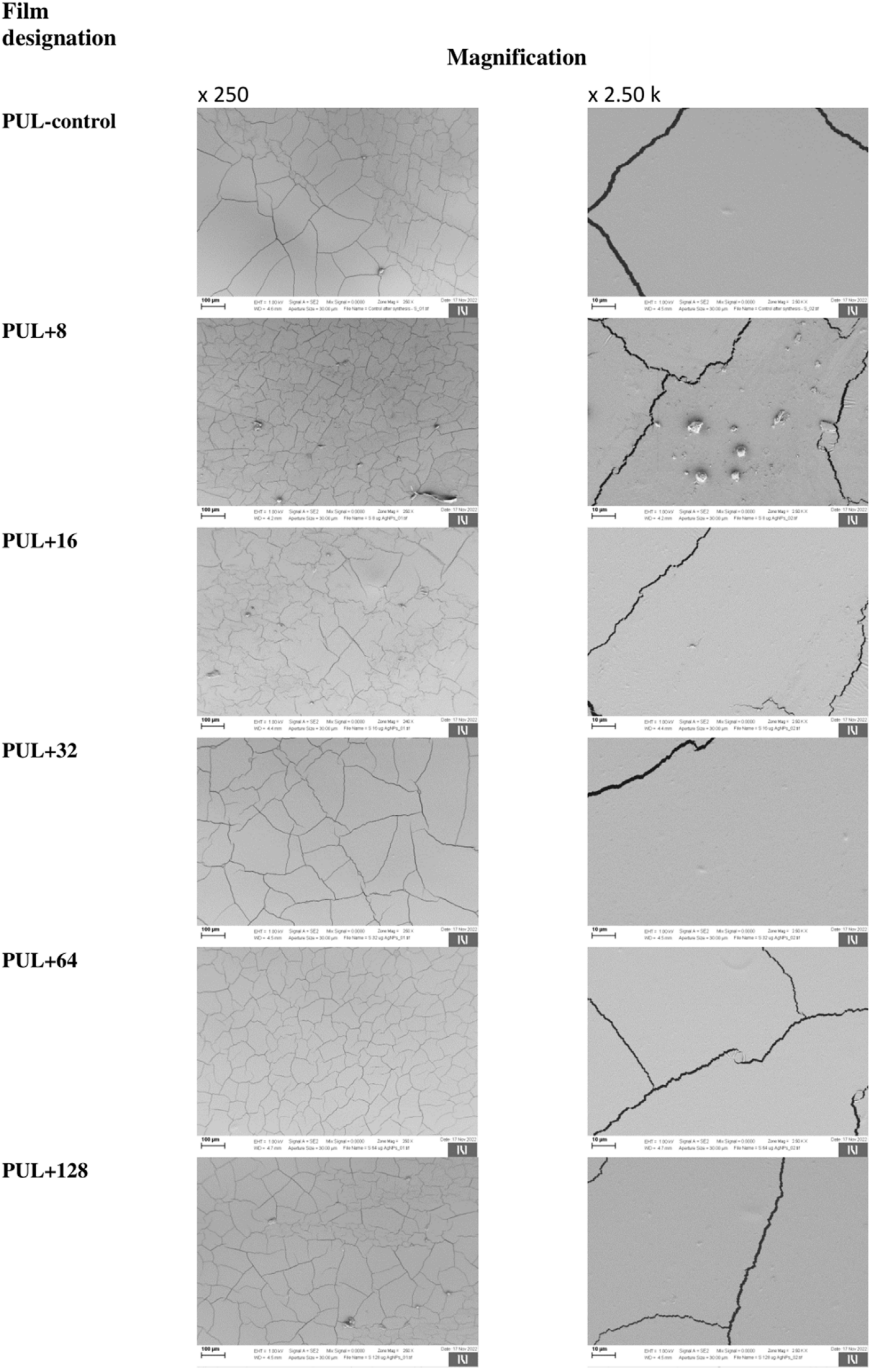
Representative images showing the structure of the films using a scanning electron microscope. PUL, pullulan film; 8–128, concentration of AgNPs in μg mL^-1^.

The surface morphology of pullulan films was investigated by scanning electron microscopy. The films had a smooth and homogenous surface without pores or holes, which confirm the good miscibility of the films and show potential food coating and preservation applications. However, SEM images of both control foil and foils enriched with different concentrations of AgNPs showed visible cracks on their surface, despite the use of glycerol as a plasticizer (Figure 9).

#### 3.2.6 UV shielding capacity

**FIGURE 10:**
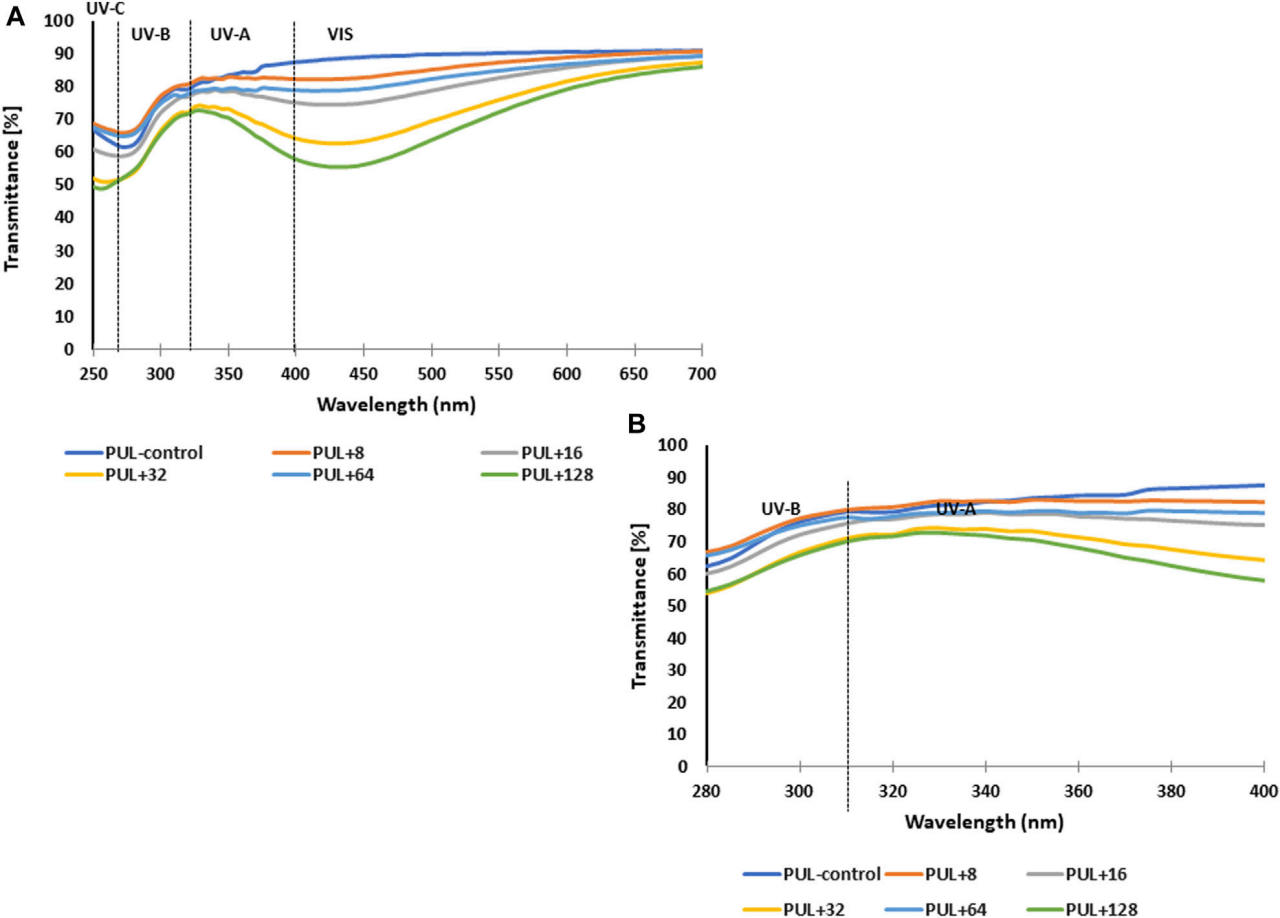
UV–Vis transmittance spectra of films prepared from synthesized pullulan from *Aureobasidium pullulans* **(A)** For better visualization, spectra in the range of 280–400 nm have been attached **(B)** PUL, pullulan film; 8–128, concentration of AgNPs in μg mL^-1^.

The UV-blocking performance of pullulan films enriched with various concentrations of AgNPs and corresponding control was characterized using the UV–Vis transmittance in the wavelength range of 250–700 nm, as shown in Figure 10. Overall, the highest light transmittance in relation to UV-A and UV-B light was shown by the control sample or samples containing the lowest concentration of mycogenic AgNPs. In contrast, the use of higher concentrations of nanoparticles in the films reduced the light transmission of both UV-B and UV-A lights.

#### 3.2.7 Degradation properties

**FIGURE 11:**
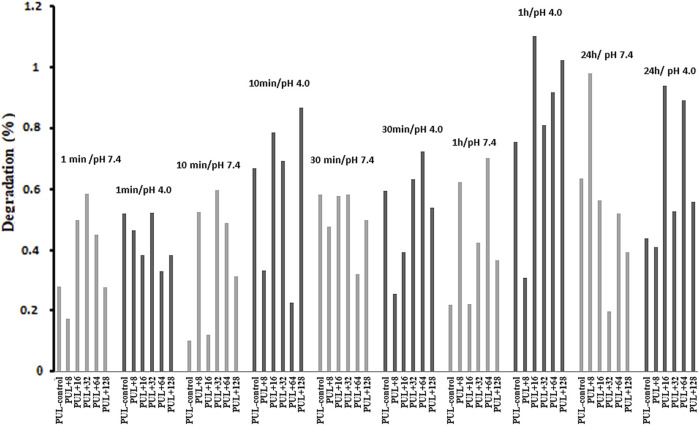
Degradation properties of pullulan films. PUL, pullulan film; 8–128, concentration of AgNPs in μg mL^-1^.

In our experiment, the degradation of pullulan films with an admixture of biogenic AgNPs was analyzed. Pullulan films were soaked at different times in solutions of different pH values, namely, pH 4.0 and 7.4. The results showed poor dissolution quality of the composite films at both pH values during 1 min, 10 min, 30 min, 1 h, and 24 h (Figure 11). For this reason, the stability of the analyzed pullulan films can be noted.

### 3.3 Antibacterial activity assays

The pullulan films enriched in 2xMIC, MIC, and 1/2MIC of AgNPs (Table 3) from the *F. culmorum* strain JTW1, based on the previously determined MIC data (Trzcińska-Wencel et al., 2023), were used against selected food-borne and reference pathogens (Supplementary Figure S2). Remarkably, for the batch of AgNPs biosynthesized for the purposes of this study, the MIC values ​​of AgNPs against tested bacteria were found to be identical. The nanocomposite films showed antibacterial activity against both food-borne and reference bacteria compared to the control samples, as shown in Table 4. Overall, higher sensitivity to nanocomposite film was found in food-borne strains than in reference strains. In addition, Gram-negative bacteria were more sensitive to the pullulan films with AgNPs compared to Gram-positive bacteria. In the case of analyzed food-borne bacteria, the highest activity of nanocomposite films was found against *L. monocytogenes*, where pullulan films were incorporated with MIC and 2xMIC of AgNPs (64 μg mL^-1^ and 128 μg mL^-1^, respectively). In contrast, films enriched with AgNPs at ½MIC value showed low activity. The antibacterial activity of foils enriched with ½MIC AgNPs (16 μg mL^-1^) (inhibition of bacterial growth under the foil test samples) was also found against food-borne *S. enterica* (Table 4; Supplementary Figure S2).

**TABLE 3:** MIC of AgNPs against food-borne and reference bacteria.

Tested bacteria	MIC (µg mL^-1^)
*Escherichia coli* ATCC 25922	32
*Escherichia coli* ATCC 8739	32
*Klebsiella pneumoniae* ATCC 700603	64
*Listeria monocytogenes* PCM 2191	64
*Pseudomonas aeruginosa* ATCC 10145	16
*Salmonella enterica* PCM 2565	32
*Salmonella infantis* SES	64
*Staphylococcus aureus* ATCC 25923	16
*Staphylococcus aureus* ATCC 6538	16

**TABLE 4:** Antibacterial activity of films prepared from pullulan synthesized using the *Aureobasidium pullulans* ATCC 201253 strain against selected bacteria.

Microorganisms	Concentrations of AgNPs			
	Control	½ MIC	MIC	2xMIC
*Escherichia coli* ATCC 25922	**-**	**-**	**-**	**+**
*Escherichia coli* ATCC 8739	**-**	**-**	**+**	**+**
*Klebsiella pneumoniae* ATCC 700603	**-**	**+**	**+**	**++**
*Listeria monocytogenes* PCM 2191	**-**	**+**	**+++**	**+++**
*Pseudomonas aeruginosa* ATCC 10145	**-**	**-**	**+**	**+**
*Salmonella enterica* PCM 2565	**-**	**+**	**+**	**++**
*Salmonella infantis* SES	**-**	**-**	**+**	**++**
*Staphylococcus aureus* ATCC 25923	**-**	**-**	**-**	**+**
*Staphylococcus aureus* ATCC 6538	**-**	**-**	**-**	**++**

Activity rating scale: zone of inhibition >1 mm, growth inhibition around and under the sample (very good activity, +++); zone of inhibition ˂1 mm, growth inhibition around and under the sample (good activity, ++); no zone of inhibition, growth inhibition under test (low activity, +); and no zone of inhibition, growth under test (no activity, -).

In turn, the pullulan-based nanocomposite films showed good or low antibacterial activity against reference bacteria (Table 4; Supplementary Figure S2). Nanocomposite films were most active against *K. pneumoniae*, following *P. aeruginosa* and *E. coli* ATCC 8739. The nanocomposite films with pullulan and AgNPs had the highest activity against *K. pneumoniae,* followed by *P. aeruginosa* and *E. coli* ATCC 8739. The weakest activity of nanocomposite films was recorded against both tested *S. aureus* reference strains and *E. coli* ATCC 25922, where the antibacterial effect was noted when 2xMIC of AgNPs was used (32 μg mL^-1^, 32 μg mL^-1^, and 64 μg mL^-1^, respectively).

## 4 Discussion

### 4.1 Synthesis of AgNPs and their characterization

Interestingly, fungi are believed to be a promising system for the extracellular synthesis of AgNPs due to the secretion of large amounts of proteins, including specific enzymes, that are involved in the reduction of silver ions and the formation of nanoparticles and their stabilization (Gudikandula et al., 2017). Moreover, fungi exhibit heavy metal tolerance and the capacity to internalize and bioaccumulate metals (Guilger-Casagrande and de Lima, 2019). The color change of the reaction mixture from pale yellow to brown after challenging the fungal extract with AgNO_3_ is typical for the formation of AgNPs (Delgado-Beleño et al., 2018). The absorption peak found at a wavelength of 428 nm is characteristic of biogenic AgNPs. Our result is in line with the findings of Osorio-Echavarría et al. (2021), who noticed a strong surface plasmon resonance (SPR) at 430 nm for AgNPs synthesized from the fungus *Bjerkandera* sp. (anamorph R1).

The AgNPs were capped with biomolecules, as they showed an intensive peak at 3,448 cm^-1^, which was assigned to the N–H stretching of the primary amine of the protein and the O–H stretching of aromatic amines (Hamedi et al., 2014; Jalali et al., 2020), at 2,927 cm^-1^ and 2,853 cm^-1^, which can be assigned to alkane C–H stretching (Ghaseminezhad et al., 2012; Wypij et al., 2021), at 1,632 cm^-1^, which corresponds to the –C=O (carbonyl) stretching vibrations in the amide bond of the proteins secreted by the fungus (Sanghi and Verma, 2009; Hamedi et al., 2014; Kavun et al., 2021), and at 1,385 cm^-1^ and 1,352 cm^-1^, which can be assigned to the C–N stretching vibrations of aromatic amines (Jain et al., 2011). The FTIR study confirmed the presence of amino acids and peptides on the surface of AgNPs that act as capping agents (Elamawi et al., 2018). It is claimed that interactions between AgNPs and proteins can occur either through free amino groups or cysteine residues in proteins and the electrostatic attraction of negatively charged carboxylate groups in the enzyme proteins (El-Batal et al., 2013; Elamawi et al., 2018; Alsamhary, 2020; Mikhailova, 2020; Wypij et al., 2022). A similar peak pattern from FTIR analyses (bands at 3,300 cm^-1^–3,400 cm^-1^, 1,630 cm^-1^–1,680 cm^-1^, and 667 cm^-1^) was shown by Osorio-Echavarría et al. (2021), who studied AgNPs synthesized from *Bjerkandera* sp. R1. It is well known that the size of nanoparticles is one of the most important parameters determining their bioactivity, including antimicrobial activity (Raza et al., 2016). For example, Xue et al. (2016) found that small-sized AgNPs, synthesized by the fungus *Arthroderma fulvum*, with an average diameter of 15.5 ± 2.5 nm and a highly uniform and narrow distribution of diameters, were effective against pathogens such as *Candida* sp., *Aspergillus* sp., and *Fusarium* sp. The nanoparticles from the *F. culmorum* strain JTW1 can also be defined as small sized with high bioactivity against a set of bacterial strains, as discussed in the following section. The zeta potential value can provide evidence of the capping agent’s efficiency in the stabilization of the nanoparticles by creating an intensive negative charge (Ashour et al., 2015). It is well known that the more negative the charge of the nanoparticles, the higher the stability of the NPs observed in the solution (Gemishev et al., 2022). In this context, AgNPs biosynthesized in the present study, with a charge of −30.1 mV, showed high stability and a very low tendency for agglomeration.

### 4.2 Properties of pullulan-based nanocomposite films

One of the important parameters related to the mechanical properties and transparency of the films is the assessment of their thickness (Khalaf et al., 2013). Although the addition of AgNPs increased the thickness of the formed nanocomposite films, they were not significantly different from the control film.

According to the work of Chu et al. (2019), the evaluation of the mechanical properties of pullulan films is very important when considering their potential application, especially in the food industry as an active packaging material. The assessment of the mechanical properties of the films provides information about their flexibility and resistance to stretching and tearing during both the food packaging process and the transport of packaging with food products (Rawdkuen, 2018). On the other hand, Soubhagya et al. (2021) studied chitosan/carboxymethyl pullulan/bioglass composite films for wound healing and found low mechanical strength and an affinity to stick to the injury surface.

It should be emphasized that proper carbon and nitrogen sources and their contents are necessary for the efficient growth of *A. pullulans* (Coltelli et al., 2020) and pullulan production, and probably higher nitrogen contents originate from the growth medium. The absorption bands, which were found in the analyzed films, at 3,305 cm^-1^ correspond to the hydroxyl group (O–H) and amine functional group (N–H), whereas the band at the 1,012 cm^-1^ region corresponds to the C–O stretching vibrations (Jeevan et al., 2012; Zemljič et al., 2020). The signals at 2,925 cm^-1^, 2,898 cm^-1^, and 1,412 cm^-1^ regions could be attributed to the vibrations of the C–H bond (Monowar et al., 2018). The absorption peaks that appeared at 1,641 cm^-1^ were attributed to the C=O functional group in the primary amide I, while those at 1,356 cm^-1^ correspond to the C–N group in the secondary amide (Zuo et al., 2019; Zemljič et al., 2020).

The hydrophilic or hydrophobic nature of films can be determined by the evaluation of water static contact angle (SCA) values. The water contact angles of hydrophilic materials are lower than 90°, while those higher than 90° indicate that the surface has a considerable hydrophobic nature (Niu et al., 2019). The settled drop technique using MiliQ water as the liquid test is used to determine the hydrophobic or hydrophilic properties of different surfaces (Slavutsky et al., 2018). Evaluating the wettability of films is very important for different applications, such as food storage or wound dressings. It is known that hydrophilic surfaces used in packaging reduce the risk of dew condensation on the surface of the film, which is the so-called anti-fog performance in contact with food (Glaser et al., 2019). On the other hand, hydrophilicity is also important for medical use, especially in wound dressing, where hydrophilic properties lead to a high absorption ability (Ghomi et al., 2019). Moreover, the roughness of the surfaces is directly related to the increase in the water contact angle, the so-called “lotus effect” (Wang et al., 2014). According to Song et al. (2019), the introduction of surface roughness is an important aspect of designing anti-wetting surfaces. For this reason, the increase in the contact angle in the analyzed films resulted from the rough surface of these samples, and this, in turn, could be associated with the formation of cracks on the surface of the films. It can be concluded that films with higher concentration of biogenic AgNPs are characterized by greater surface roughness. Additionally, the type of additives may affect the contact angle value of the polymer films, as shown in the studies by Luís et al. (2020a). They showed that pullulan enriched with rockrose essential oil increased the water contact angle of pullulan film from 65° to 74°, thus increasing its hydrophobicity. However, Zhao and coauthors (2019) analyzed the water contact angle of pure pullulan and pullulan–lactoferrin blend films and observed that the additive lactoferrin at concentrations of 0.3% and 0.6% did not affect the contact angle value of 59°, as observed for the pure pullulan film. The authors concluded that the surface hydrophilicity of pullulan–lactoferrin blend films was independent of lactoferrin concentration (Zhao et al., 2019). Yang et al. (2019) reported that a lack of pores on the surface of chitosan films increases the quality of coated products. The presence of cracks is undoubtedly related to the increase in the contact angle and surface roughness, which were mentioned previously. It is well known that the use of plasticizers reduces porosity and affects the smoothing of the film surface. Plasticizers are an important additive that improves flexibility, maintains the integrity of the films, and prevents the formation of cracks in the polymer matrix (Garcia et al., 2000; Mohammed et al., 2023). Glycerol, a low-molecular-weight molecule, is a commonly used plasticizer that weakens the intermolecular interactions between the polysaccharide chains, resulting in a less-compact structure of the polymer with enhanced flexibility (Tarique et al., 2021; Concórdio-Reis et al., 2022). Moreover, glycerol can effectively improve the plasticity of the film as it is a good humectant (Concórdio-Reis et al., 2022). However, Narayanan and Lee (2016) suggested that fractures can be caused by shrinkage of the surface layer during the drying process in a hot air oven and loss of water. Additionally, fractures can occur as a result of the presence of samples in a vacuum environment during SEM testing. In our work, pullulan-based films showed discrete particles/slight lumps separated from each other, which could be formed due to a lack of homogeneity in the film-forming solution, differences in hydrophilicity, or petty impurities that penetrated during the pouring of the films. Overall, according to Olewnik-Kruszkowska et al. (2022), the presence of different concentrations of additives such as polyethylene glycol (PEG) and a chloroformic extract of propolis in produced films based on polylactide (PLA) significantly influences their roughness. They showed that with an increase in the concentration of propolis in the films, an increase in the number of fissures, depressions, cracks, and lumps on the surface was observed. For this reason, it can be concluded that the addition of biogenic AgNPs can change the morphology and texture of the films compared to the control. Hernandez-Tenorio and Giraldo-Estrada (2022) analyzed the surface of pullulan film produced by *A. pullulans* ATCC 15233 and pullulan-acetate film using SEM and showed homogeneous morphology with a compact surface structure of the former one, while small particles on the surface of the latter one were observed.

One of the very important functions of coatings or films, especially in terms of food storage, is light transmission (Xing et al., 2019). Lozano-Navarro et al. (2017) noticed that radiation in a certain wave range may contribute to the oxidation of packaged food ingredients and the deterioration of their color. According to Wang et al. (2020), films with low light transmission can be a good barrier to UV light, which in turn can help extend the shelf life of products that are packaged. In this context, it is important to limit light transmission by packaging films into the food. The reduced transparency in the UV regions can indicate good UV shielding capacity of films (Jancic et al., 2021). The obtained results suggest that the addition of AgNPs to pullulan films can improve the optical properties of the films and affect the safety of stored food. Similarly, Zhang et al. (2021) reported that biodegradable composite films based on pullulan/carboxymethyl cellulose/nano-TiO_2_ showed significantly decreased transmittance with an increase in titanium nanoparticle content.

Interestingly, the pullulan films with AgNPs were characterized by low degradation properties that are beneficial for potential applications, e.g., in food packaging. Moreover, the mechanical strength and limited degradation of pullulan nanocomposites make them suitable for use in *in vivo* studies. Fricain et al. (2013) studied a scaffold composed of pullulan and dextran with hydroxyapatite particles (nHA) in the bone healing process in various animal models such as mice, goats, and rats. They noticed that such a composite matrix stimulated bone cells and bone formation.

### 4.3 Antibacterial activity of pullulan-based nanocomposites

Nowadays, pullulan-based nanocomposite films play a crucial role as antimicrobial agents against various pathogens (Rai et al., 2021). Compounds/substances with bioactive properties are added to coatings and films to reduce, inhibit, or delay the development of microorganisms (Rai et al., 2021; Wypij et al., 2023). Pullulan films enriched with polycyclic peptides and essential oils demonstrate potential antibacterial activity against both clinical and food-borne pathogens (Hassan and Cutter, 2020). Likewise, various metal nanoparticles play an important role in many applications, including the pharmaceutical sector, healthcare, and the food storage industry (Ganduri et al., 2016). Among the different types of metallic nanoparticles, AgNPs can be highlighted for their broad-spectrum antimicrobial potential (Guilger-Casagrande and de Lima, 2019). Therefore, pullulan-based nanocomposite films can be used in wound infections caused by bacteria and fungi and in packaging and food storage (Li et al., 2020).

In the present study, the lower activity of AgNPs against Gram-positive bacteria can be due to a higher percentage of peptidoglycan content in their cell wall when compared with the Gram-negative bacteria (Wypij et al., 2021).

It is known that AgNPs and the released Ag ions may adsorb on the bacterial surface and destabilize the cell membrane, leading to leakage of protons that disrupt the electron transport chain, decrease ATP synthesis, and cause cell death. The incorporation of AgNPs into bacterial cells and further release of ions generate oxidative stress, as silver ions can act as cofactors for bacterial enzymes that are involved in the production of reactive oxygen species (ROS) such as hydroxyl radicals, superoxides, and hydrogen peroxide. It consequently damages cell proteins, lipids, and DNA. Silver ions can denature small ribosomal subunits and affect protein synthesis. Moreover, AgNPs, by binding to sulfur and phosphorus groups in DNA, disturb the transcription and translation processes (Pangli et al., 2021; Wypij et al., 2021; 2022).

Pullulan coatings impregnated with antimicrobial compounds such as nisin, lauric arginate, and thymol (Hassan and Cutter, 2020) and nanoparticles, namely, AgNPs and ZnONPs (Khalaf et al., 2013), have gained interest and importance due to their promising applications in different sectors (Rai et al., 2021). Khalaf et al. (2013) studied the antibacterial activity of pullulan films enriched with commercial AgNPs and ZnONPs (with a diameter of 100 and 110 nm, respectively) and 2% oregano and rosemary essential oils (EOs) against *L. monocytogenes* and *S. aureus* and reported that the latter was more sensitive to the pullulan films with both NPs and EOs than the former one. Moreover, Gniewosz and Synowiec (2011) analyzed the antibacterial effect of pullulan films containing thymol against *Bacillus subtilis* ATCC 6633, *S. aureus* ATCC 25923, *Salmonella enteritidis* ATCC 13076, and *E. coli* ATCC 25922 and showed that Gram-positive bacteria were more sensitive than Gram-negative bacteria to the films enriched with thymol. Recently, Paneysar et al. (2022) reported the antibacterial activity of a temperature-responsive film fabricated from pullulan-g-pNIPAM and impregnated with two different concentrations (15 ppm and 30 ppm) of chemically synthesized AgNPs as a potential dressing for wounds against both Gram-positive *S. aureus* and Gram-negative *E. coli*. They proposed these nanocomposite films, constructed from temperature-responsive polymers that release AgNPs when the temperature of the wound exudate is slightly higher than normal, as a novel therapeutic material for the management of non-healing wounds. Moreover, Wang et al. (2022) demonstrated that a complex wound dressing developed from hyaluronic acid-grafted pullulan succinate (HA-st-Pu) with chitosan (CS) had good antibacterial activity against *E. coli* and *S. aureus* and accelerated skin wound repair. Mao et al. (2022) studied chitosan–hyaluronic acid–pullulan composite film wound dressings for antibacterial activity and found that they exhibited a certain antibacterial capability against *E. coli* and *S. aureus* and wound healing. In addition, Duan et al. (2020) evaluated curcumin-grafted hyaluronic acid-modified pullulan polymers as a functional wound dressing material. The study showed bactericidal activity against *E. coli* and *S. aureus*, and the analyzed film is considered a promising and safe formulation for accelerating skin wound healing.

## 5 Conclusion

To sum up, novel nanocomposite films were successfully prepared based on pullulan synthesized from *A. pullulans* ATCC 201253 and incorporated with biogenically synthesized AgNPs from the extract of *F. culmorum* strain JTW1. The impregnation of AgNPs into pullulan film did not significantly affect the thickness, whereas a change in color intensity from colorless (controls) to slightly brown was observed with increasing concentrations of AgNPs. The pullulan-based nanocomposite films demonstrated antibacterial activity against both food-borne and reference bacterial pathogens. Moreover, the highest activity of the prepared films was observed against *L. monocytogenes*. In turn, Gram-negative bacteria were more sensitive to the pullulan films with AgNPs than Gram-positive bacteria. In this context, the obtained results are very promising and indicate that pullulan-based nanocomposite films with the addition of mycogenic AgNPs could be used in biomedicine, as medical products for wound healing, and for food packaging and storage and natural active packaging to protect food against food-borne bacteria. However, controlled release of AgNPs (slow-release) from such films is required to prevent significant cytotoxic effects on human cells.

## Data Availability

The original contributions presented in the study are included in the article/Supplementary Material; further inquiries can be directed to the corresponding authors.
