# Supplementary material for: Pullulan-based films impregnated with silver nanoparticles from the Fusarium culmorum strain JTW1 for potential applications in the food industry and medicine
Source: Front Bioeng Biotechnol. 2023 Aug 7;11:1241739. doi: 10.3389/fbioe.2023.1241739 (PMC10441246; doi:10.3389/fbioe.2023.1241739)
Supplement: Supplementary file 1 [file Presentation1.PPTX]

## Slide 1
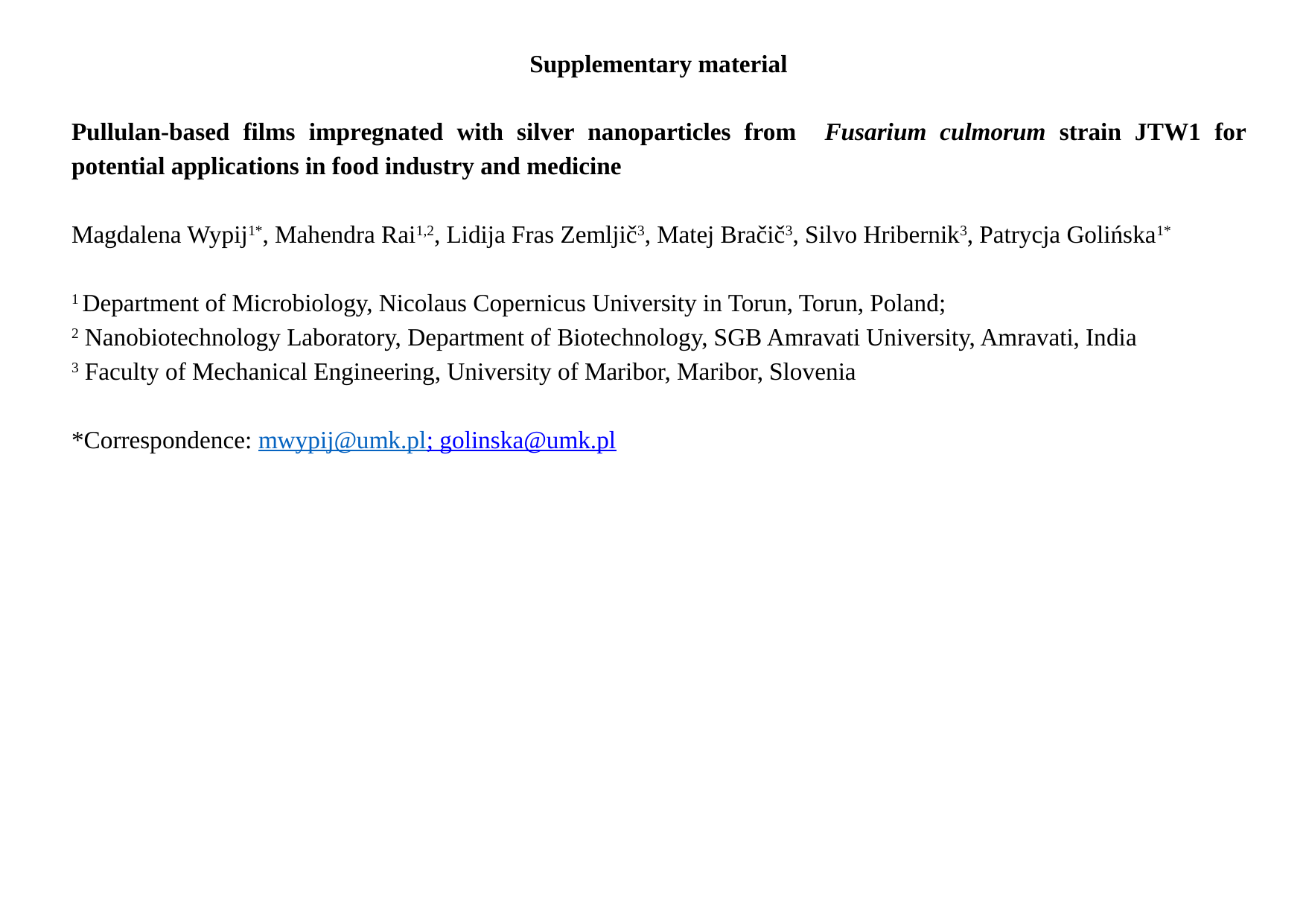

Supplementary material
Pullulan-based films impregnated with silver nanoparticles from Fusarium culmorum strain JTW1 for potential applications in food industry and medicine
Magdalena Wypij1*, Mahendra Rai1,2, Lidija Fras Zemljič3, Matej Bračič3, Silvo Hribernik3, Patrycja Golińska1*
1 Department of Microbiology, Nicolaus Copernicus University in Torun, Torun, Poland;
2 Nanobiotechnology Laboratory, Department of Biotechnology, SGB Amravati University, Amravati, India
3 Faculty of Mechanical Engineering, University of Maribor, Maribor, Slovenia
*Correspondence: mwypij@umk.pl; golinska@umk.pl

## Slide 2
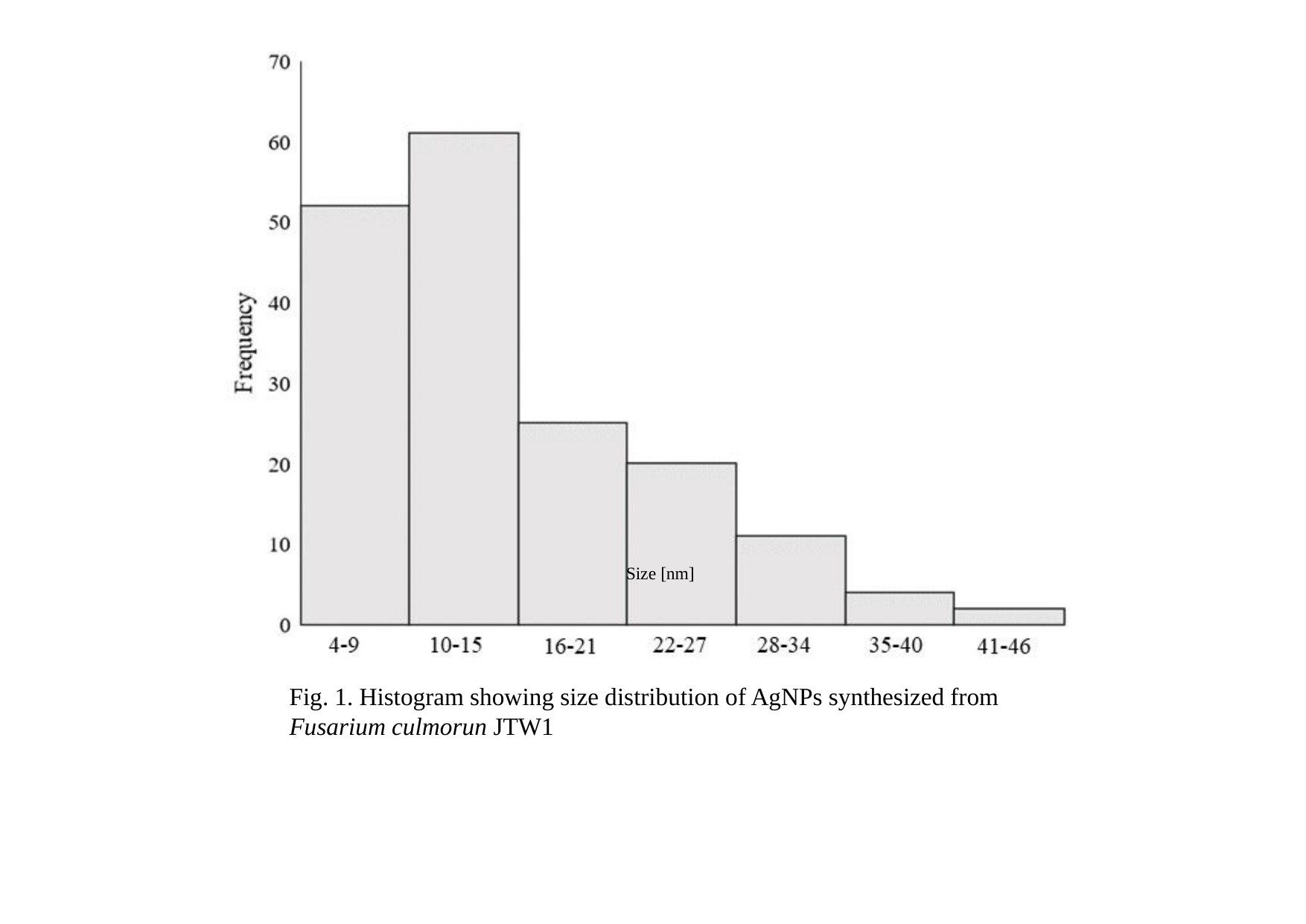

Size [nm]
Fig. 1. Histogram showing size distribution of AgNPs synthesized from Fusarium culmorun JTW1

## Slide 3
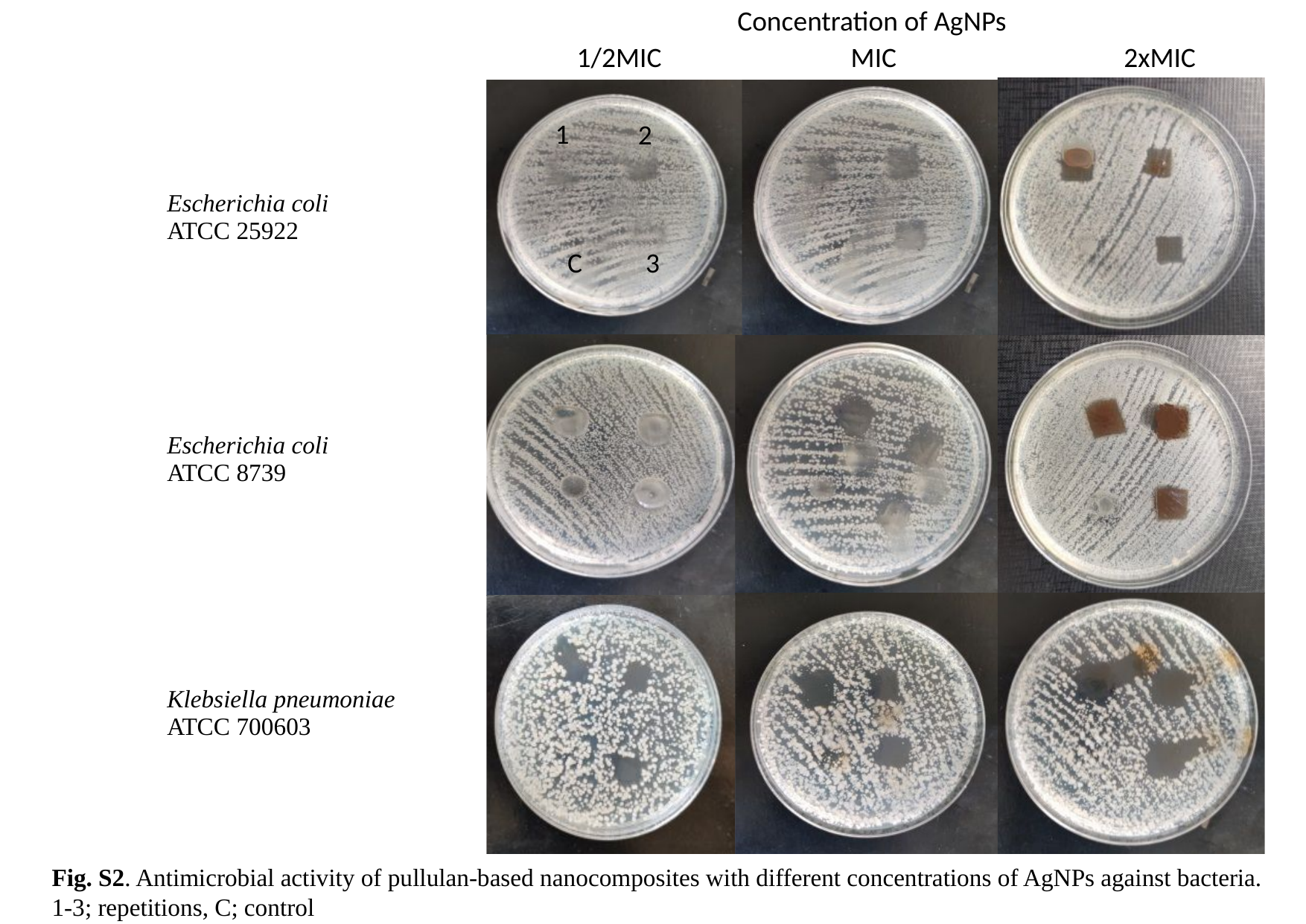

Concentration of AgNPs
1/2MIC MIC 2xMIC
1
2
C
3
1
2
2
1
| Escherichia coli Escherichia coli ATCC 25922 |
| --- |
| Escherichia coli ATCC 8739 |
| Klebsiella pneumoniae ATCC 700603 |
C
3
C
3
Fig. S2. Antimicrobial activity of pullulan-based nanocomposites with different concentrations of AgNPs against bacteria.
1-3; repetitions, C; control

## Slide 4
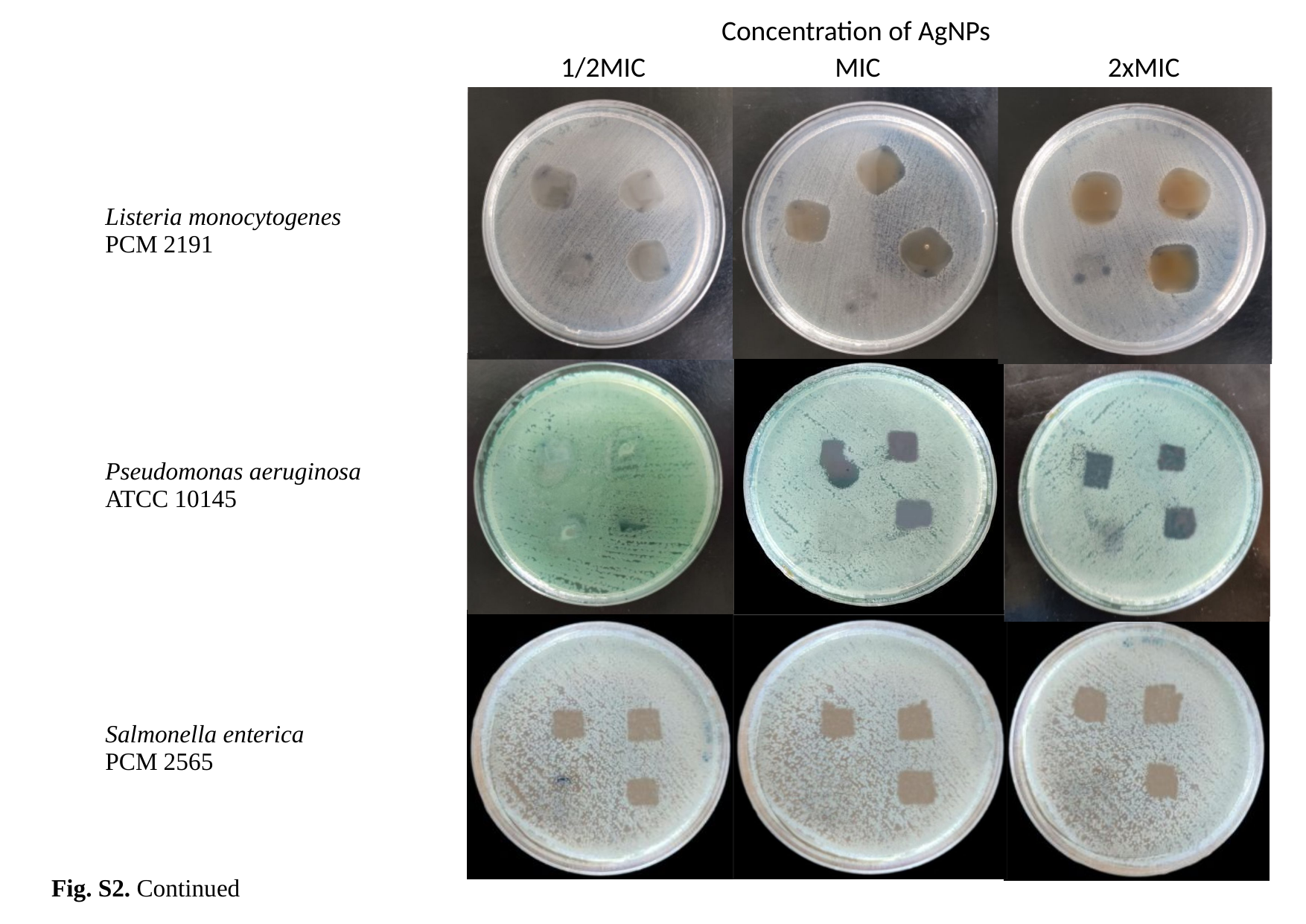

Concentration of AgNPs
1/2MIC MIC 2xMIC
| |
| --- |
| Listeria monocytogenes PCM 2191 |
| |
| |
| Pseudomonas aeruginosa ATCC 10145 |
| |
| Salmonella enterica PCM 2565 |
| |
2
1
3
C
Fig. S2. Continued

## Slide 5
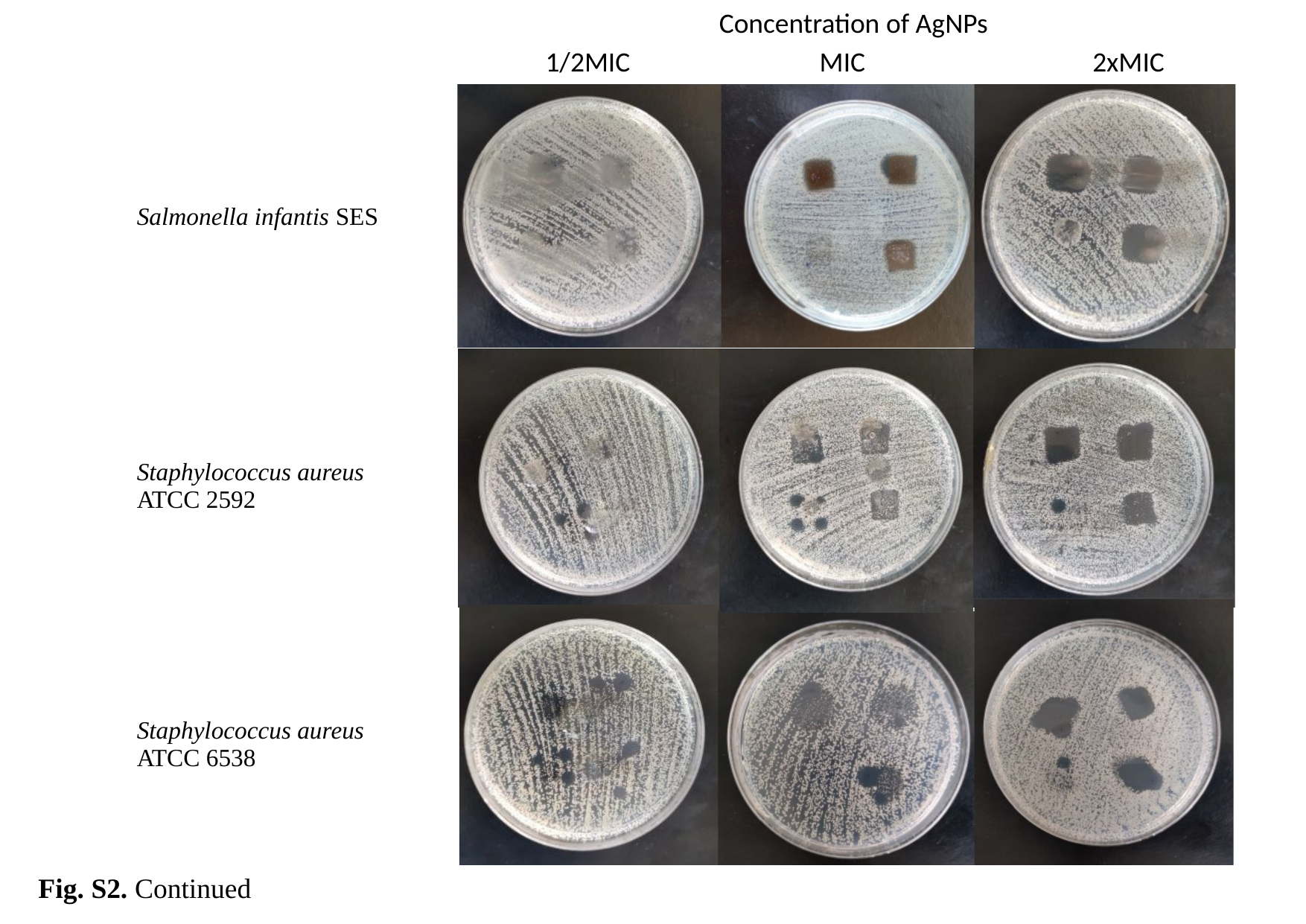

Concentration of AgNPs
1/2MIC MIC 2xMIC
| Salmonella infantis SES |
| --- |
| |
| Staphylococcus aureus ATCC 2592 |
| Staphylococcus aureus ATCC 6538 |
Fig. S2. Continued
